# High-Quality Draft Genome Sequence of a *Rickettsiales* Bacterium Found in *Acropora tenuis* Coral from Okinawa, Japan

**DOI:** 10.1128/MRA.00848-20

**Published:** 2020-11-25

**Authors:** Keigo Ide, Yohei Nishikawa, Masato Kogawa, Eisuke Iwamoto, Ashok Zachariah Samuel, Yoshikatsu Nakano, Haruko Takeyama

**Affiliations:** a Department of Life Science and Medical Bioscience, Waseda University, Tokyo, Japan; b Computational Bio Big-Data Open Innovation Laboratory, National Institute of Advanced Industrial Science and Technology, Tokyo, Japan; c Research Organization for Nano and Life Innovation, Waseda University, Tokyo, Japan; d Tropical Biosphere Research Center, University of the Ryukyus, Okinawa, Japan; e Okinawa Marine Science Support Section, Research Support Division, Okinawa Institute of Science and Technology Graduate University, Okinawa, Japan; f Institute for Advanced Research of Biosystem Dynamics, Waseda Research Institute for Science and Engineering, Tokyo, Japan; Indiana University, Bloomington

## Abstract

*Rickettsiales*-like organisms are important for the survival and functioning of corals, prompting an investigation of their complete genomes. Earlier reports of the genomes of these organisms remain incomplete. Here, we report a novel draft genome of *Rickettsiales* bacterial strain SESOKO1, found in *Acropora tenuis* coral, using single-cell genome technology.

## ANNOUNCEMENT

*Rickettsiales*-like organisms live symbiotically associated with invertebrates (e.g., corals and shellfish) (1, 2). A majority of *Rickettsiales*-like organisms coexisting with several corals are unculturable bacteria (3) and are known to cause white band disease (4, 5). A clear understanding of the full genomes of these bacteria is lacking due to a paucity of available full-genome sequences (6). In this context, the single-cell isolation method could be valuable for mapping uncontaminated genomic information. In this study, we report a high-quality draft genome, obtained using droplet-based single-cell genomics, of a *Rickettsiales*-like organism that was collected from *Acropora tenuis* coral (7, 8).

An *Acropora tenuis* coral branch and 100 ml of seawater were collected from Sesoko Island, Okinawa, Japan (26.629911N, 127.857914E). The seawater was filtered through a membrane filter (0.22 μm, MF-Millipore) and exposed to UV light for ∼30 min. The coral branch was kept in 5 ml of this treated seawater, crushed using a disposable scalpel, and then kept on ice for 5 min. The mixture was filtered, and the supernatant was collected (1.5 ml) and centrifuged at 8,000 × *g* for 5 min. After three repeated washes, the bacterial fraction was resuspended to 50 μl. Single-cell whole-genome amplification was performed using the droplet-based method (SAG-gel) (9). Briefly, we encapsulated bacterial single cells in monodispersed picoliter-sized immiscible droplets (encapsulation rate of 0.1 cell/droplet). After encapsulation of the cells in droplets, the cells were subjected to cell lysis and multiple displacement amplification (MDA) with the REPLI-g single-cell kit (Qiagen, Inc., Valencia, CA, USA) at 30°C for 3 h. Then, the droplets containing amplified DNA were isolated with a fluorescence-activated cell sorting (FACS)-based technique. These isolated DNA-containing droplets were subjected to a second round of MDA.

Whole-genome sequencing was conducted by 2 × 75-bp paired-end sequencing with the Illumina MiSeq platform. Default parameters were used for all software unless otherwise specified. Genome assembly was performed by SPAdes v.3.13 (10). Ten host mitochondrial sequences were removed as contamination using BLASTn v.2.9.0+ with the nucleotide database (11). Assembled genome annotation was performed using Prokka v.1.14.5 (12). Genome quality assessment was conducted using CheckM v.1.1.2 (13). The assembled genome was taxonomically classified with GTDB-Tk v.1.1.1 (14). The 16S rRNA gene sequence was assigned to a RefSeq record with a BLASTn v2.9.0+ search (11).

Statistics for the constructed genome are presented in Table 1. We have now designated the genome as a bacterium in the *Rickettsiales* order (GTDB-Tk output). This 16S rRNA sequence is closely similar to *Anaplasma phagocytophilum* strain JM (Refseq; NC_021880.1, 86.13%).

**TABLE 1:** Genome sequence statistics for *Rickettsiales* bacterial strain SESOKO1

Characteristic	Value(s)
No. of contigs	199
Largest contig length (bp)	99,919
Total genome length (bp)	1,084,225
Coverage (×)	876
*N*_50_ (bp)	19,338
GC content (%)	41.3
No. of coding sequences	1,030
No. of tRNAs	37
No. of rRNAs (5S, 16S, 23S)	3 (1, 1, 1)
Completeness (%)	95.1
Contamination (%)	0.47

### Data availability.

The assembled genome was deposited in DDBJ/ENA/GenBank under the accession number SAMD00233765. The raw read data are available under BioProject number PRJDB10112 and DDBJ Sequence Read Archive (DRA) accession number DRR235466.
